# Promoting theory of mind and emotion understanding in preschool settings: an exploratory training study

**DOI:** 10.3389/fpsyg.2024.1439824

**Published:** 2024-08-06

**Authors:** Ilaria Grazzani

**Affiliations:** Department of Human Sciences for Education “R. Massa”, University of Milano-Bicocca, Milan, Italy

**Keywords:** theory of mind, emotion understanding, social understanding, social and emotional learning, training study, preschoolers

## Abstract

**Introduction:**

This new exploratory study is part of a larger ongoing follow-up project. Its specific aim was to verify whether an innovative European Program, primarily designed to enhance children’s social and emotional learning, led to gains in theory of mind and emotion understanding when implemented in preschool settings.

**Methods:**

Thirty-four children (mean age: 56.4 months; *SD*: 10.1; range: 40–70 months) participated in the study. They were randomly and equally divided into a training group and a control group. The training sample completed eight linguistic-conversational activities drawn from the Program, in groups of 5 to 6 children, over 8 weeks. The activities were based on listening to stories and/or watching videos and then thinking and talking about the inner world (thoughts and emotions) of the story characters as well as the participants’ own inner states. During the training phase, the children in the control group engaged in drawing or free play activities. At both the pre-test and post-test phases of the study, all the children completed a language test, a battery of theory of mind (ToM) tasks (including ‘change of location’ and ‘unexpected content’ tasks), and the Test of Emotion Comprehension which evaluates nine components of emotion understanding (EU). The validated national versions of the tests were administered in all cases.

**Results:**

Significant differences were identified between the training and control groups. Indeed, the participants in the Program training activities, which were based on conversational exchanges between an adult and a group of children, as well as among the children themselves, outperformed the control participants on both overall theory of mind and overall emotion understanding. A more detailed analysis showed that the training group outperformed the control group in relation to both specific components of EU and the ‘change of location’ ToM task.

**Discussion:**

The results of this exploratory study suggest that the Program is effective at enhancing preschoolers’ social understanding and thus merits implementation in preschool settings.

## 1 Introduction

Social understanding, which informs everyday exchanges within interpersonal relations, is the ability to interpret ourselves and others in psychological terms, and specifically as persons with inner states such as intentions, desires, emotions, beliefs, false beliefs, and other complex mental experiences. Human beings begin the long process of trying to get to grips with the social world *early* in life. Indeed, recent studies have emphasized that implicit elements of this ability can already be observed during the second year of life or even before, as in the case of infants’ spontaneous helping conducts (Buttelmann et al., 2009), their displays of empathy (Bischof-Kohler, 2012), and their looking behaviors, interpreted as an indicator of their attribution of false beliefs to others (Baillargeon et al., 2018).

Indeed, social understanding encompasses both theory of mind (ToM) and emotion understanding (EU) (Tompkins et al., 2018; Grazzani and Ornaghi, 2022; Grazzani and Conte, 2024). Theory of mind has primarily been studied in relation to children’s developing ability to understand that they and others possess epistemic mental states such as beliefs and false beliefs. Wellman (2014) outlined the main phases in ToM development, proposing a model whereby the child sequentially acquires an appreciation of desires, beliefs, and first- and second-order false beliefs between the ages of 2 and 8 years approximately. Harris and colleagues (Pons et al., 2004) investigated the development of emotion understanding, describing children’s progressively more sophisticated comprehension of the nature, causes, and regulation of emotions between the ages of 3 and 11 years. While the literature offers well-established models of how ToM and EU generally develop as a function of age, it remains crucial to identify the factors that determine variations in children’s theory of mind (ToM) and emotion understanding (EU) and shape both typical and atypical patterns of development (Montgomery et al., 2023).

Although recent studies have shown that forms of ToM and EU already feature during the pre-verbal developmental phase, language continues to be one of the most intensively investigated factors in relation to the development of social understanding (Tompkins et al., 2018). The complexity of language is well known to philosophers, linguistics, and psychologists, who – in relation to the development of ToM and EU – have focused their attention on one or more of its various dimensions, ranging from syntax to semantics and pragmatics. Indeed, metanalyses (Milligan et al., 2007) and systematic reviews (Beaudoin et al., 2020) have shown that language matters for social understanding; for example, it may facilitate the transition from implicit to explicit theory of mind and emotion understanding, given that language allows children to talk about invisible inner states and heightens their awareness of their own mental experiences.

This study is informed by the social constructivist position that adult-child conversations around mental states promote children’s social understanding (Ornaghi et al., 2011; Slaughter and de Rosnay, 2017; Carpendale and Lewis, 2020). Research in this domain not only corroborates the importance of mental state language *per se* but also the role of conversation that focuses on, and directly uses, this particular kind of lexicon. Among the large number of studies that have examined this topic, many have homed in on the interaction between parent and child. Symons et al. (2006) showed that the more a mother, during her everyday conversational interactions with her child, uses mentalistic nouns, adjectives, and verbs, the better the child’s later performance on false belief tasks. Adrian et al. (2005) found that the frequency and variety of the cognitive and emotional terms used by mothers during storybook reading and conversations with their preschool children were positively correlated with the latter’s performance on false belief tasks. Similarly, Aram et al. (2013) showed that the cognitive and social and emotional understanding of preschool children was significantly enhanced when their parents read them stories that featured mental state lexicon and then discussed the story characters’ thoughts and social interactions with them. This significant association has been borne out by more recent studies, including a German longitudinal study (Ebert et al., 2017) with preschool children in which the effect of the participants’ socio-economic background was also controlled for, as well as a cross-cultural study by Taumoepeau et al. (2019) that compared samples of Australian and Iranian mothers. The latter authors only identified a significant correlation between false belief understanding and maternal mental-state talk in the group of Australian mothers, who while reading and discussing an unillustrated story with their children, used more cognitive mental state terms and referred more frequently to their own inner states.

## 2 Promoting social understanding in the preschool context

While there are numerous studies on parent–child interaction and conversation in family surrounding mental states and inner experience, less research has been carried out in extrafamilial contexts, as shown in a meta-analysis by Hofmann et al. (2016). The study of conversational interaction about mental states between educators/teachers and children at nursery or kindergarten can concern both the adult-individual child interaction and the interaction between an adult and a small group of children. The empirical focus of the present work is on this second possibility.

Conversation between an adult and a small group of children can in principle foster the development of abilities that are key for social understanding. In the course of group conversations, children are encouraged to listen to the utterances of others (e.g., statements, comments, questions, and answers), to put themselves in the shoes of others, and to compare their own point of view with that of others. This kind of activity can enhance the perspective-taking ability that is crucial to understanding the social world and to recognizing that – with respect to oneself – another person may hold different or similar intentions, perceptions, thoughts, beliefs, emotions, feelings, needs, motives, and information (Carpendale and Lewis, 2020).

To date, few studies have explored how children’s ToM and EU may be enhanced via programs based on activities with small groups of children in preschool educational settings. We define ‘programs’ here as structured interventions with accompanying guidelines that can help adults in educational contexts (e.g., educators, teachers, education specialists, psychologists, and so on) to conduct targeted activities with a view to improving children’s social understanding. In a pioneering study focused on the Italian cultural context and preschool children, Ornaghi et al. (2011) implemented a two-month intervention in kindergartens, during which 3- and 4-year-olds were read stories enriched with mental state terms. After listening to a story, the participants took part in guided language games and conversations aimed at stimulating their use of a variety of mental state terms. As compared to the control group, the intervention group displayed significant gains in their theory-of-mind and metacognitive vocabulary comprehension. This intervention informed the development of a structured program based on language games around mental terms, including ‘to think’, ‘to be afraid’, ‘to decide’, ‘to remember’ and so on (Grazzani and Ornaghi, 2022). In addition, Grazzani and Ornaghi (2011) and Ornaghi et al. (2015) found similar results with children aged 3 to 5 years who participated in emotion language games and subsequently displayed significant gains in their emotion understanding and use of emotional lexicon. However, to date, no intervention program has been associated with either of these two studies. In a study by Mori and Cigala (2019), small groups of preschool children were trained in perspective taking. The significant outcomes obtained prompted the development of a program for schools that targets multiple dimensions of perspective taking, from the perceptive to the cognitive dimension (Mori and Cigala, 2021). The program offers a wide variety of structured activities involving movement, drawing, and dramatization. The promotion of theory of mind in preschool age is in continuity with that enhanced in primary school context. Bianco et al. (2019), for instance, showed that conversation about mental states allowed children between 7 and 8 years of age to outperform in ToM skills children belonging to the control group. Similar findings were described in Bianco et al. (2021) with a large sample of students who showed a significant training effect on advanced-ToM and metacognition abilities. A validated program for improving theory of mind in primary school children is presented in the volume by Lecce and Bianco (2018) which includes precise guidelines for teachers.

**Figure 1:**
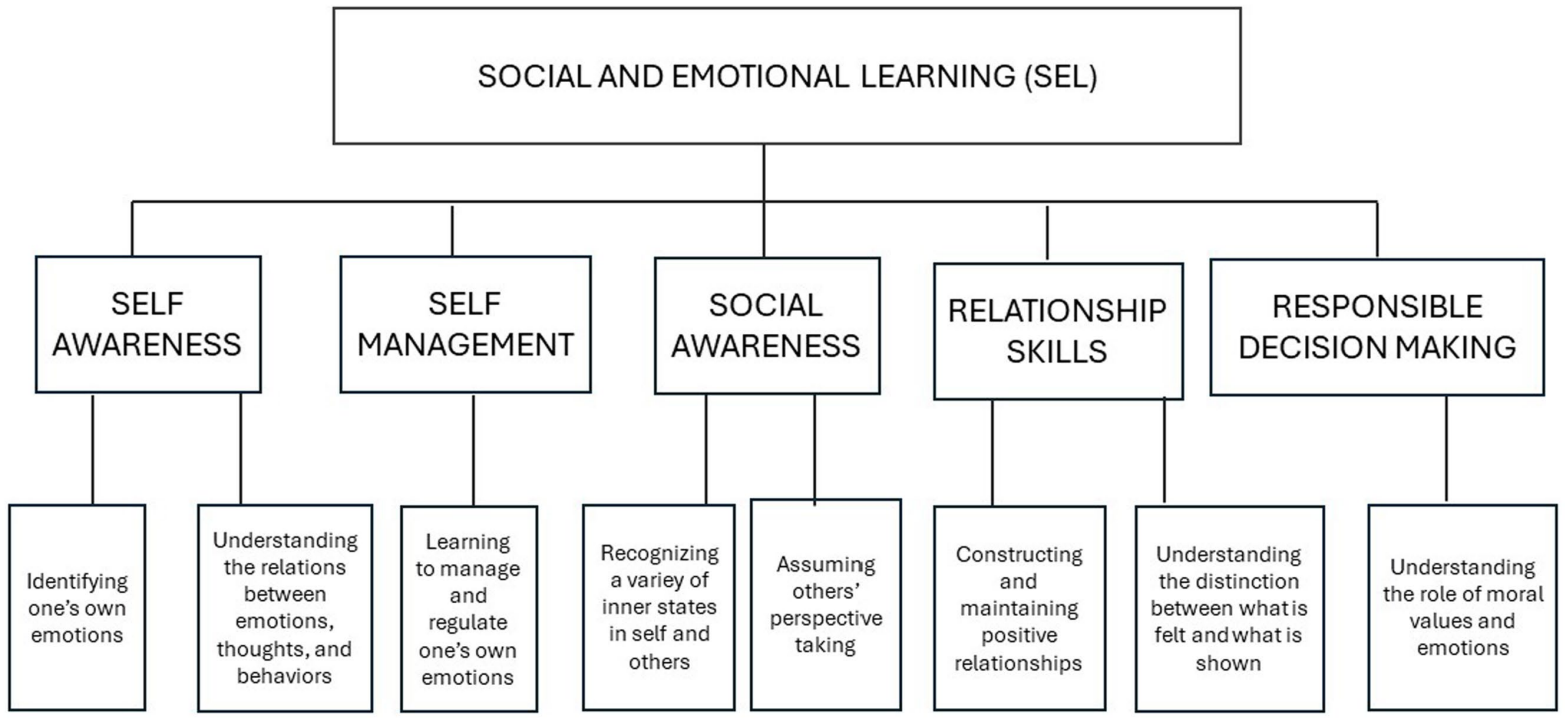
SEL components ant the related trained activities.

The PROMEHS program/curriculum, which is partly based on conversational activities, has recently been validated as an effective means of enhancing the social and emotional learning (SEL) of preschool, primary, and secondary school students (Cefai et al., 2022). The Program targets the five main components of SEL (Durlak et al., 2015; CASEL-Collaborative on Academic Social Emotional Learning, 2020; Cavioni et al., 2020a): self-awareness (the ability to identify one’s own thoughts, emotions, and needs), self-management (the ability to regulate one’s own thoughts, emotions, and behaviors), social awareness (the ability to understand another person’s point of view, thoughts, and emotions), relationship skills (the ability to establish and maintain positive social relationships) and responsible decision making (the ability to make informed decisions). Each of the five components corresponds to a specific set of competences or sub-skills well described by the CASEL’s framework. For example, self-awareness includes awareness of one’s own thoughts and emotions and how these relate to one’s own actions; social awareness concerns the perspective-taking process and empathic behavior; relationship skills encompass the ability to coordinate with others, which presupposes the capacity to recognize their needs, intentions, thoughts, and so on (Mahoney et al., 2021). Figure 1 illustrates the relationship between the five SEL components on the one hand, and the activities implemented during the training which targeted these subskills on the other hand. It can be seen that these sub-skills evoke the above definition of social understanding in terms of theory of mind and emotion comprehension, a definition that underlines the awareness of one’s own thoughts and emotions as well as the distinction between one’s own and others’ internal states (Wellman, 2014).

During the original experimental evaluation of the PROMEHS Program (Cefai et al., 2022), its effectiveness with preschoolers was only verified indirectly by asking the children’s teachers to complete the SSIS-SEL Brief Scales by Elliott et al. (2020). As noted in Conte et al. (2023), this instrument now validated in Italian (Cavioni et al., 2023) comprises 20 items to be rated on a 4-point Likert scale ranging from 1, “not true” to 4, “very true.” It was used to indirectly assess participants’ SEL competences before and after the experimental intervention. As such, it did not provide direct evidence of the Program’s impact on children’s skills.

## 3 The present study

In light of the background outlined above, we conducted a new training study with two groups of participants: an experimental group (or training group) and a control group. The study had two main aims:

1. Given that the effectiveness of PROMEHS Program had not previously been tested using direct measures, in this study we set out to implement part of the program via a targeted training intervention with a new, smaller sample of children, and to assess the impact of the training by administering direct measures;
2. Given that – as noted above – the components of SEL overlap with those of social understanding, in this study we set out to evaluate the effectiveness of the PROMEHS Program by administering direct measures of the participating children’s theory of mind and emotion understanding. Differences between the training and control groups at post-test would corroborate the hypothesis that the Program activities under study, which were specifically devised to promote SEL, may usefully be included in interventions targeting preschoolers’ social understanding as well, that is to say, their theory of mind and emotion understanding abilities.

Based on the results of earlier training studies conducted using a conversational approach (for a review: Tompkins et al., 2018), we expected to find significant differences between the experimental group (or training group) and the control group following the intervention.

## 4 Method

### 4.1 Participants

The participants in this study were 34 children (16 girls; mean age at the beginning of the study = 56.4 months, range = 40–70 months; *SD* = 10.1). Evenly divided into two groups (a training and a control group), the children were enrolled at two nursery schools in an urban area of a Northern Italian region. The head teachers agreed for the schools to participate in the study because of the potential benefits for their preschools and teachers. All the children were native Italian speakers. The inclusion criteria for this study were: (1) the children were required to be aged between 40 and 72 months, and (2) the parents were required to provide informed consent for their children’s participation in the study. The parents, who were of medium socioeconomic status, attended a presentation of the study, and then signed the informed consent forms for their children. This approach to obtaining informed consent was in line with the Declaration of Helsinki principles. Participants were free to withdraw from the study at any time. The study was conducted in conformity with the recommendations of the University of Milano-Bicocca Ethics Committee.

### 4.2 Measures

The research instruments selected were validated measures and appropriate to the age of the participants. We followed Denham’s recommendations (Denham et al., 2010) who suggested evaluating the effectiveness of evidence-based SEL programs through tasks directly administered to children. Both before and after the training phase, the children completed a battery of tests, in counterbalanced order, in a quiet and familiar room at their nursery school. More specifically, the following tests were administered by a member of the research team who had spent familiarization time getting to know the children beforehand.

#### 4.2.1 Peabody picture vocabulary test

The PPVT (Dunn and Dunn 2007) was administered both to verify that the two groups were starting out with equivalent levels of linguistic ability and to verify that none of the participants displayed strongly atypical language development. We use the Italian standardized version of the test (Stella Pizzoli and Tressoldi, 2000). It evaluates the receptive vocabulary of children between 3 and 12 years and consists of 180 cards, each containing four numbered illustrations among which the child is asked to indicate the one that corresponds to the word called out by the examiner. Scoring was carried out following the standard procedure, with 1 point assigned for each correct answer and 0 for each wrong answer. The reliability coefficient was *α* = 0.70.

#### 4.2.2 Test of emotion comprehension

The TEC devised by Pons and Harris (2000) assesses emotion comprehension in 3- to 11-year-olds. It encompasses nine components (Pons et al., 2004), namely: the recognition of facial expressions of emotions; the understanding of, respectively, the impact of situational causes on emotions, the role of desires in emotions, the role of beliefs in emotions, the impact of memory on emotions, the distinction between outwardly expressed and privately felt emotions, and the effect of morality on emotions; the awareness that emotions may be regulated using cognitive control strategies; and, finally, an appreciation of concurrent mixed feelings. In the current study, we deployed the standardized Italian version (Albanese and Molina, 2008). The TEC assesses emotion understanding by presenting vignettes in which a gender-matched protagonist encounters simple to complex situations eliciting different emotional responses. After each vignette, the child is asked to indicate how the protagonist feels, by choosing among four illustrations of faces representing different emotional states. For each group of items testing an individual component, a score ranging from 0 to 1 is awarded. These scores are then summed to obtain a Total TEC score, which ranges from 0 to 9. The scoring system was defined and applied in strict accordance with the guidelines of Pons and Harris (2000) and subsequent recommendations by Cavioni et al. (2020a,b). The reliability coefficient was *α* = 0.73.

#### 4.2.3 ToM battery

This measure consisted of two first-order false-belief tests: (a) a ‘false-belief location change task’ consisting in the Italian adaptation of the classic “Sally and Ann” story (Liverta Sempio et al., 2005); (b) a false-belief unexpected content task consisting in the Italian adaptation by Liverta Sempio et al. (2005). For each task, the children were awarded scores of 1 for the correct answer and 0 for the wrong answer. Scores for the battery were summed to yield a possible maximum total score of 2. The reliability coefficient was *α* = 0.80.

### 4.3 The program and training procedure

Following the pre-test phase, the training phase was implemented. The children in the training group participated in eight activities lasting approximately 45 min each, offered once a week over a period of eight weeks. The training activities were selected from the PROMEHS Program (for further information, see Grazzani et al., 2020) as a function of the children’s age and of the aim of the study, which was to foster competences relating to the SEL components. Figure 1 shows how the components of SEL are related to the respective target activities and competences.

The training was conducted by a member of the research team with weekly supervision by the project leader. The trainer spent a week developing a relationship with the children before initiating the intervention. Each training session involved a small group of children and was divided into two parts. First, the trainer read aloud to the children a story drawn from the PROMEHS Program (e.g., ‘The three little pigs’). Second, the children took part in conversational activities guided by the trainer, who followed the guidelines provided in the manual of the Program. The children were prompted to actively participate in the conversation by answering questions and recounting and sharing their own experiences, emotions, and thoughts. During the conversational activities, the trainer emphasized the subjective nature of mental states and encouraged the children both to discuss their own perspectives and to adopt the perspectives of others. The children in the control group simply listened to the stories and then participating in unstructured drawing activities. All the teachers who participated in the study agreed to be trained after the post-test phase. Supplementary Appendix A1 outlines a sample of activities from the Program whose aim is to foster the ability to “identify and name basic emotions,” “learn to regulate one’s own emotions,” “assume others’ perspective taking,” “construct and maintain positive relationship” and “understand the role of moral values and emotions.”

## 5 Results

All the data analyses in this exploratory study were conducted using the software IBM SPSS Version 29. Before analyzing the efficacy of the training, standard data-cleaning procedures were conducted. No missing values were detected. Anticipating a medium effect size based on prior research in this domain, we set the desired *power level a priori* at 0.70. The analysis indicated that a minimum of 16 participants per group condition would be required to obtain 95% statistical power in detecting the expected effect. The data were matched by code to combine the pre- and post-test scores; all the children received scores for both tests.

The results section comprises two subsections outlining descriptive statistics for all study measures and the impact of the training on children’s theory of mind and emotion comprehension, respectively. Please recall that, given the linguistic-conversational nature of the training, the language measure was administered amongst other reasons with a view to identifying any atypical patterns of language development.

### 5.1 Descriptive statistics

Descriptive statistics (*n* = 34) at Time 1 and Time 2 for the variables under study are reported in Table 1. These include the means and standard deviations, both before and after the training phase, of the following: age in months, language ability as assessed by the Peabody Test, emotion understanding as evaluated by the TEC (including each of the nine components mentioned in the Measures section), and theory of mind as assessed via the ToM battery with the change of location and unexpected content tasks. Correlations were calculated using the pre-test data. Age in months was positively and significantly associated with language (*r* = 0.70, *p* < 0.001), Total TEC (*r* = 0.63, *p* < 0.001) and Total ToM (*r* = 0.50, *p* = 0.004); language was significantly associated with both Total TEC (*r* = 0.64, *p* < 0.001) and Total ToM (*r* = 0.41, *p* = 0.02); there was a significant association between EU and ToM, and more specifically between Component 9 of EU (an appreciation of concurrent mixed feelings) and Total ToM (*r* = 0.37, = *p* = 0.04).

**Table 1:** Means and standard deviations of all variables both before and after the training phase.

	MEAN (pre and post)	SD (pre and post)
Age in months	56.48–58.84	10.158–10.080
Languge ability (Peabody)	53.45–60.32	23.490–25.718
Emotion understanding (TEC)		
TEC - Component 1	0.94–0.97	0.250–0.180
TEC - Component 2	0.81–0.74	0.402–0.445
TEC - Component 3	0.52–0.71	0.508–0.461
TEC - Component 4	0.52–0.65	0.508–0.486
TEC - Component 5	0.35–0.71	0.486–0.461
TEC - Component 6	0.29–0.42	0.461–0.502
TEC - Component 7	0.48–0.55	0.508–0.506
TEC - Component 8	0.06–0.23	0.250–0.425
TEC - Component 9	0.52–0.61	0.508–0.495
TEC - Total score	4.48–5.58	1.730–2.277
Theory of mind (Battery of tasks)		
ToM (Change of location)	0.39–0.58	0.495–0.502
ToM (Unexpected content)	0.32–0.45	0.475–0.506
ToM - Total score	0.71–1.03	0.824–0.875

### 5.2 The effectiveness of the training

At pre-test, the training and control groups did not differ significantly in relation to any of the dependent variables, namely language ability (*p* = 0.537), emotion understanding (*p* = 0.333), and theory of mind (*p* = 0.567). No differences emerged as a function of gender; therefore, this variable was not included in the subsequent analyses.

To test the impact of the training activities, a repeated measures multivariate analysis of variance (Manova) was carried out. The independent variables were Time (pre-test and post-test) and Group Condition (training vs. control); Time was a within-participant variable whereas Group Condition was a between-participant variable. The dependent variables at the two time points were language ability, emotion understanding (Total TEC), and theory of mind (Total ToM). Effect sizes were calculated using partial eta-squared (*η_p_^2^*) values.

Table 2 presents the means and standard deviations at Time 1 (pre-test) and Time 2 (post-test) as a function of Group Condition (training vs. control group).

**Table 2:** Pre- and post-test means and standard deviations for all variables by group condition.

	Training group		Control group	
	Pre-test	Post-test	Pre-test	Post-test
Age in months	57.67 (8.9)	60.07 (8.8)	55.38 (11.9)	57.69 (11.2)
Languge ability (Peabody)	56.20 (21.2)	64.47 (25.3)	50.88 (25.8)	56.44 (26.3)
**EU (TEC)**				
TEC - Component 1	0.93 (0.25)	1.00 (0.0)	0.94 (0.25)	0.94 (0.25)
TEC - Component 2	0.87 (0.35)	0.93 (0.25)	0.75 (0.44)	0.56 (0.51)
TEC - Component 3	0.53 (0.51)	0.93 (0.25)	0.50 (0.51)	0.50 (0.51)
TEC - Component 4	0.47 (0.51)	0.67 (0.48)	0.56 (0.51)	0.63 (0.50)
TEC - Component 5	0.47 (0.51)	0.80 (0.41)	0.25 (0.44)	0.63 (0.50)
TEC - Component 6	0.33 (0.48)	0.53 (0.51)	0.25 (0.44)	0.31 (0.47)
TEC - Component 7	0.47 (0.51)	0.80 (0.41)	0.50 (0.51)	0.31 (0.47)
TEC - Component 8	0.7 (0.25)	0.27 (0.45)	0.6 (0.25)	0.19 (0.40)
TEC - Component 9	0.67 (0.48)	0.80 (0.41)	0.38 (0.50)	0.44 (0.51)
TEC – Total Score	4.80 (1.6)	6.73 (1.4)	4.19 (1.7)	4.50 (2.4)
**ToM (Battery of tasks)**				
ToM 1 Change of loc.	0.33 (0.48)	0.80 (0.41)	0.44 (0.51)	0.38 (0.50)
ToM 2 Unexpected content	0.47 (0.51)	0.60 (0.50)	0.19 (0.40)	0.31 (0.47)
ToM – Total Score	0.80 (0.86)	1.40 (0.73)	0.63 (0.80)	0.69 (0.87)

Standard deviations are in parentheses.

Concerning the effect of the training on emotion understanding (Total TEC), there was a significant Time x Group Condition interaction, Wilks’s λ = 0.80, *F* = 6,99, *p* = 0.01, *η_p_^2^* = 0.194. The univariate test showed that the training group outperformed the control group, with the former displaying a significantly greater gain in their global emotion understanding, *F* = 9,57, *p* = 0.004, *η_p_^2^* = 0.248. from pre- to post-test.

Concerning the impact of the training on ToM (Total score), there was a significant Time x Group Condition interaction, Wilks’s *λ* = 0.74, *F* = 10.04, *p* = 0.004, *η_p_^2^* = 0.257. The univariate test showed that the training group outperformed the control group, in that the former displayed a significantly greater gain from pre- to post-test in their global theory of mind, *F* = 5,98, *p* = 0.02, *η_p_^2^* = 0.171. The interaction Time x Group Condition was not significant for language ability, Wilks’s λ = 0.98, *F* = 0,33, *p* = 0.57, *η_p_^2^* = 0.011.

Analyses for the Time factor revealed that the control group displayed a significant pre- to post-test increase in language ability, *t* (15) = 8,58, *p* < 0.001, TEC Total score, *t* (15) = 7,432, *p* < 0.001, and ToM Total score, *t* (15) = 3,149, *p* = 0.007. Similarly, the training group displayed a significant pre- to post-test increase in language ability, *t* (15) = 9,866, *p* < 0.001, TEC Total score, *t*(15) = 18,140, *p* < 0.001, and ToM Total score, *t* (15) = 9,866, *p* < 0.001.

**Figure 2:**
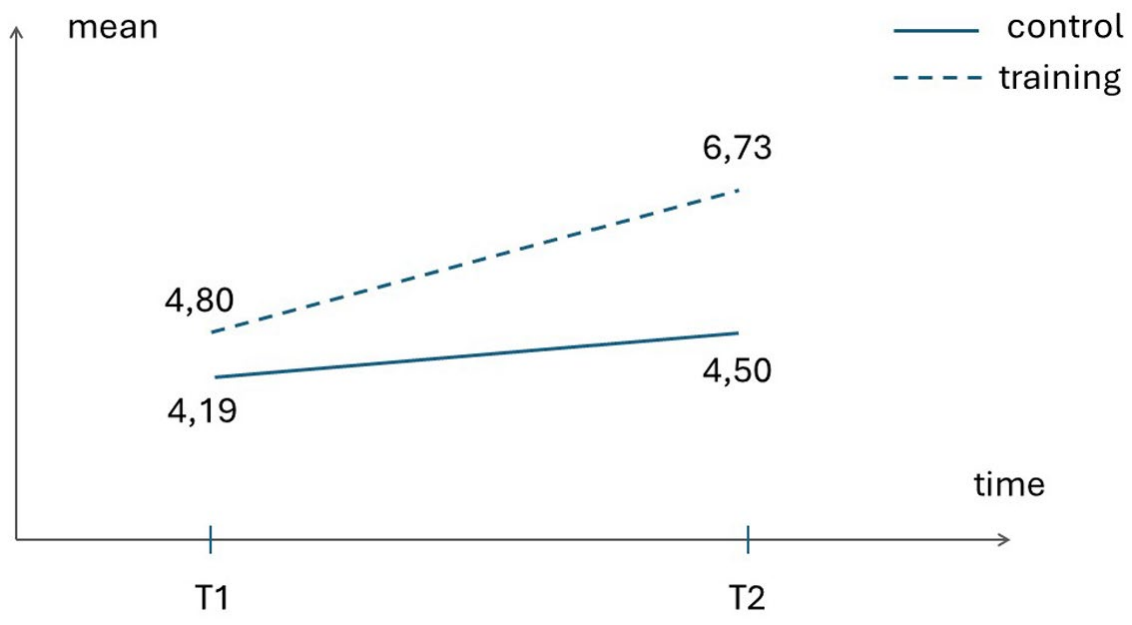
Control and Training groups’ scores on emotion understanding at pre-test (Tl) and post-test (T2).

**Figure 3:**
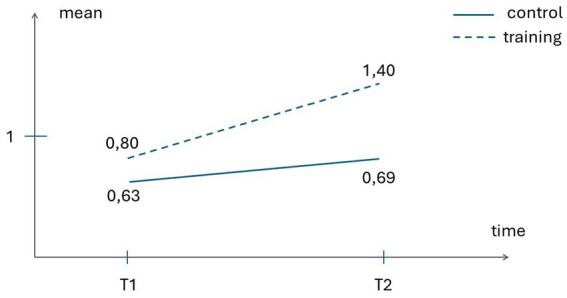
Control and Training groups’ scores on theory of mind at pre-test (Tl) and post-test (T2).

Figures 2, 3 illustrate the significantly greater improvement of the training group – as compared to the control group – from pre-test (Time 1) to post-test (Time 2) in global EU as well as in global ToM.

When we analyzed the impact of the training on specific components of the TEC, we identified significant effects on Component 2, understanding the impact of situational causes on emotions (*F* = 6,33, df = 1, *p* = 0.01), Component 3, understanding the role of desire in emotions (*F* = 8,54, df = 1, *p* = 0.007), Component 7, the effect of morality on emotions (*F* = 9,14, df = 1, *p* = 0.005), and Component 9, the appreciation of concurrent mixed feelings (*F* = 4,65, df = 1, *p* = 0.03). Finally, analysis of the impact of the training on the individual tests in the Theory of Mind Battery showed that the training group improved significantly more on the ‘change of location’ test (*F* = 6,59, df = 1, *p* = 0.01) than did the control group.

## 6 Discussion

This study built on the previous research of Conte et al. (2023) which was designed to verify the effectiveness of the PROMEHS program in promoting preschoolers’ social and emotional learning. In the study by Conte et al. (2023), however, the effectiveness of the program was only tested using indirect measures, namely teacher-report questionnaires (Elliott et al., 2020). Identifying appropriate direct measures and administering them to a fresh sample of children remained an open challenge. In the current study, one objective was thus to evaluate a part of the PROMEHS Program for preschoolers via direct measures, in line with the recommendations of Denham et al. (2010).

Furthermore, given the link between the components of SEL and those of social understanding as illustrated in Figure 1, we selected direct measures of theory of mind and emotion understanding to investigate the effectiveness of the program. The proposal to evaluate the impact of a SEL curriculum also through theory of mind measures is innovative as compared to the existing findings. We expected that we would find differences in post-test performance between the children who had participated in the experimental (training group) and those in the control group who had only listened to stories, watched videos, or engaged in unstructured drawing activities without participating in the conversational activities. The data analysis shows that at Time 2 (post-test), the mean test performance of both groups of children had improved relative to Time 1. However, further analyses confirmed our research hypothesis, showing that the children in the training group displayed significantly greater gains in both theory of mind and emotion understanding than did the children in the control group. This effect was more powerful with respect to emotion understanding (effect size: 0.248) than with respect to theory of mind (effect size: 0.171). Both effect sizes were modest, yet in line with those found in similar past studies (e.g., Ornaghi et al., 2015). Plausibly, larger effect sizes might be found by increasing the size of the research sample.

PROMEHS’ relatively greater impact on EU may be explained by the characteristics of the Program itself, which includes a high proportion of activities that engage the emotional sphere and specifically target skills such as the recognition of emotions and their causes, and the ability to regulate ongoing emotional experience. The data analysis showed that particularly significant gains were displayed in components of EU that directly featured in the training activities, such as understanding the role of desires in emotions as well as the effect of morality on feelings.

It may be concluded that regularly conducting conversational activities with small groups of children over an approximately two-month period favored the development of the skills required to perform well in the tests, such as the ability to adopt the perspective of others and compare it to one’s own, as well as the ability to identify the characteristics of different emotions and to grasp their relationship with manifest behaviors. The training intervention was designed to maximize conversational exchanges between the adult and the children as well as among the children themselves. In the course of the training, this objective was increasingly more fully attained, as the children became more and more familiar with this kind of activity and increasingly more adept at actively contributing to the conversations. This implied being able to progressively talk more about themselves and others, rather than about the characters in the stories, and to contribute in an original and non-imitative way to the conversational exchanges. To this regard, consider this brief language exchange among children involved in a conversation about anger regulation, in which they manage the exchange without the need of teacher intervention. *Teacher*: What can we do to make the anger go away? *Paolo*: We can drink some water. *Anna*: eh …but that does not make the anger go away. *Paolo*: at best [it works] when you cry and are very angry. *Giulia*: you can at least breath *Paolo*: you can stay cool.

Listening to the stories and to the stimulus questions and input of the trainer prompted the children to actively join in conversations about inner states and to deploy a variety of terms from the psychological lexicon, including emotional, cognitive and volitive terms. Through language and conversation, the children were becoming more acutely aware of thoughts and emotions as causes of actions and also, vice versa, actions as causes of emotions and thoughts. Moreover, the conversational activities encouraged the children to engage in processes of metacognitive reflection about their own and others’ internal states and to recognize the difference between the external causes of emotions and internal causes such as memories and desires. The children were also encouraged to take the perspective of the story characters, and to explicitly state what they would have done and how they would have felt had they been in these characters’ shoes. They were also prompted to explicitly discuss how to deal with complex situations such as feeling too sad or extremely angry, or when others display strong negative emotions. The children were also invited to think about good and polite behaviors (their own and others) that made them feel good (such as cooperating, helping, sharing toys, consoling) and to contrast these with behaviors that made them feel bad (e.g., fighting, hitting, snatching toys).

Finally, it should be noted that this program was initially implemented to foster social and emotional learning hence its efficacy in enhancing children’s social understanding could not be taken for granted. Furthermore, the competences associated with the five components of SEL targeted by the Program are not exclusively related to social understanding, given that they also include prosocial conduct, decision making, and problem solving. Hence, a program devised to promote SEL is not entirely comparable to a program for the development of social understanding understood as ToM and EU. The key findings of the present study are therefore that implementing the PROMEHS SEL Program with preschool children is indeed linked with gains in social understanding as well, while the direct measures administered effectively captured these improvements.

## 7 Limitations, strengths, and implications

The limitations of this study should be noted. First, this was an exploratory study with a small number of participants. More studies and more data are required to corroborate these interesting preliminary findings on the effectiveness of this program in enhancing preschoolers’ theory of mind and emotion understanding.

Second, broader studies are required to take into account other variables that have not been considered here, such as the socio-economic status of the participants, possible differences in the effectiveness of the program as a function of age, alternative control group activities (for example a group that does drama instead of conversation activities), and longitudinal follow-up data. In addition, the size of this sample did not allow us to delve into the role of other factors such as the presence of siblings, which deserve attention in future research.

Third, drawing on the most recent theorizing surrounding adult-child conversations about mental states (Tompkins et al., 2022; Farrell et al., 2023), more detailed investigations are required to maximize the impact of programs such as that implemented in the present study. In this regard, the nature of the children’s contributions to the conversational exchanges (for example, appropriate vs. off-topic comments) on the one hand, and the adult’s linguistic style (for instance, tending to use open-ended questions versus closed questions) deserve more in-depth scrutiny. This knowledge could then be applied when training education practitioners to implement the program. Importantly, most studies that have drawn on the social constructivist perspective informing our work here have focused on the interaction between one adult (e.g., a parent) and one child rather than between an education practitioner and a group of children.

Despite these limitations, the findings of this study suggest that the PROMEHS Program has a significant impact in terms of effectively fostering not only children’s understanding of emotions, as might be hoped of a program that intentionally targets social and *emotional* learning, but also their theory of mind abilities. We therefore believe that this program, which is already available in numerous European languages, can produce positive effects when used in preschool settings by appropriately trained practitioners. In these educational contexts, access to structured programs with materials and guidelines can greatly facilitate the implementation of targeted educational activities with children here. In addition, this kind of program could be particularly useful for those children who have poor socio-emotional skills or show a delay in linguistic development also due to an immigration background, in line with the results found by Conte et al. (2023) with children with an atypical profile. Listening to stories, the encouragement to speak through stimulating questions, listening to the classmates’ comments and responses create, in fact, a favorable context for the development of cognitive, linguistic and socio-emotional skills. In sum, this study contributes to the existing body of applied research that is informed by the concept that social understanding may be taught in kindergarten (Grazzani and Brockmeier, 2019), a privileged setting for exploring how children come to understand their social world and for ‘validating’ the best educational practices for enhancing this understanding.

## Data availability statement

The raw data supporting the conclusions of this article and the material of the Program will be made available by the author as appropriate.

## Ethics statement

The study was conducted in conformity with the recommendations of the University of Milano-Bicocca Ethics Committee. Written informed consent to participate in this study was provided by the participants’ legal guardian/next of kin.

## Author contributions

IG: Conceptualization, Data curation, Formal analysis, Funding acquisition, Methodology, Project administration, Supervision, Writing – original draft, Writing – review & editing.
