# Supplementary material for: Promoting theory of mind and emotion understanding in preschool settings: an exploratory training study
Source: Front Psychol. 2024 Aug 6;15:1439824. doi: 10.3389/fpsyg.2024.1439824 (PMC11333321; doi:10.3389/fpsyg.2024.1439824)
Supplement: Supplementary file 1 [file Data_Sheet_1.pdf]

Appendix 1: A sample of training activities from the PROMEHS Program (simplified).

SEL COMPONENT: SELF-AWARENESS

Definition: Self-awareness is the ability to recognize one's own emotions and thoughts, and how these affect one's behavior.

Target competence: Knowing how to identify and classify basic and complex emotions

Training phases

- The teacher reads the story "The Three Little Pigs" for the children (or alternatively, shows them a YouTube video of the story).
- Language and conversational activities. After reading the story, the teacher asks the children:
    1. How do you think the little pigs felt while they were building their houses? Why?
    2. How do you think the first two little pigs felt after the wolf blew their houses down? Why?
    3. How do you think the wolf felt when he wasn't able to blow down the third house? Why?
    4. What emotions can you recognize in "The Three Little Pigs?"
- The teacher produces the emotion cards and asks each child in turn to express the emotion represented on a card; the children put themselves in the shoes of the characters in the story they just listened to, identifying with their emotions.
- The teacher asks a child, for example, to act the part of an angry little pig, and then asks the other children, who do not know what instruction their classmate has been given, to guess what emotion is being represented

SEL COMPONENT: SELF MANAGEMENT

Definition: Self-management is the ability to regulate one's own thoughts, emotions, and behaviors

Target competence: learning to regulate one's own emotions

Training phases

- The teacher reads the story on 'anger'.
- Language and conversational activities. After reading the story, the teacher asks the children:

    1.What does the protagonist feel? What emotions and thoughts?

    2.What does the protagonist do to no longer feel so much anger?

    3.What would you do if you felt so much anger?

4. How can you help a friend who is very angry?

SEL COMPONENT: SOCIAL AWARENESS

Definition: Social awareness is the ability to understand another person's point of view, thoughts, and emotions

Target competence: Assuming others' perspective taking

Training phases

- The teacher and the children watch a cartoon - with no words - on the theme of kindness (YouTube video). The topic is the welcoming of a new classmate.
- Language and conversational activities. After watching the cartoon, the teacher asks the children:

  1.What can you do to make this child feel accepted?

  2.How would you feel afterwards?

  3.What could the classmate think about your behavior?

  4.What could the new classmate desire arriving in the new class?

SEL COMPONENT: RELATIONSHIP SKILLS

Definition: the ability to coordinate with others, which presupposes the capacity to recognize their needs, intentions, thoughts

Target competence: constructing and maintaining positive relationship

Training phases

- The teacher tells the children the story of one of her friends. The topic is the friendship.
- Language and conversational activities. The teacher asks the children:

  1.Which of you, children, has a friend you want to talk about?

  2.What do you like most about your friend?

  3.How can you understand that your friend needs your help?

  4.How do you feel when you argue with your friend? How do you make peace?

SEL COMPONENT: RESPONSIBLE DECISION MAKING

Definition: the ability to make careful and constructive choices towards oneself and others

Target competence: understanding the role of moral values and emotions

Training phases

- The teacher tells the children the story “The empty vase”.
- Language and conversational activities. The teacher asks the children:

  1.Why did the emperor choose Ping as his successor?

  2.What do you think when someone tells a lie?

  3. Why is it important respect the rules in the school?

  4. How do you feel when you don’t respect the class rules?
